# Overview of the First Phylogenomics Conference

**DOI:** 10.1186/1471-2148-7-S1-S1

**Published:** 2007-02-08

**Authors:** Hervé Philippe, Mathieu Blanchette

**Affiliations:** 1 Canadian Institute for Advanced Research, Centre Robert Cedergren, Département de Biochimie, Université de Montréal, 2900 Boulevard Édouard-Montpetit, Montréal, Québec, H3T 1J4, Canada; 2 McGill Centre for Bioinformatics, McGill University, 3775 University Steet, Montréal, Québec, H3A 2B4, Canada

## Abstract

The First Phylogenomics Conference was held in Ste-Adèle (Québec, Canada) in March 2006. Selected papers appear in this special issue of *BMC Evolutionary Biology*. Here, we give an introduction to the field and provide an overview of the articles presented in this issue.

## Introduction

The newly arising discipline of phylogenomics owes its existence to the revolutionizing progress in DNA sequencing technology. The number of completely sequenced genomes is already high and increases at an ever-accelerating pace. The newly coined term phylogenomics (portmanteau word for phylogenetics and genomics) comprises several areas of research at the interface between molecular biology and evolution. The main issues are: (1) using genomic data to infer phylogenetic relationships and gain insights into the mechanisms of molecular evolution, and (2) using multi-species comparisons and phylogenetics to infer putative functions for DNA or protein sequences. The word phylogenomics was first introduced in 1998 in the context of an "approach to the prediction of gene function" for genome-scale data [1], and soon after in the context of phylogenetic inference [2]. The majority of publications on phylogenomics deal with the use of phylogenies to make more sense out of genomic and proteomic data (see [3] for review). However, the use of data at the genomic scale to reconstruct the phylogeny of organisms also attracted a lot of interest recently (see [4] for review).

The First Conference on Phylogenomics was held in March 2006 in Ste-Adèle (Québec, Canada), with the goal of creating a synergy between the two phylogenomics communities. These communities rarely meet, so we created this conference to help bridging the gap between their respective scientific endeavours. Indeed, the knowledge of the accurate species phylogeny increases the quantity and quality of functional information that can be inferred. Conversely, knowledge of gene function and the other selective constraints is primordial to improve tree reconstruction methods. The conference was attended by more than 140 participants, with backgrounds in evolution, genomics, molecular biology, microbiology, bioinformatics, and mathematics. It featured 12 invited speakers, 33 contributed presentations, and more than 30 posters.

Although the themes of the talks were very diverse, one unifying factor besides phylogeny and genomics emerged: the development and use of complex models of evolution, allowing sound statistical inference in a probabilistic framework. The importance of such an approach has been emphasized for many years [5-7], and the field appears now to be mature. The growing importance of models does not decrease the importance of an adequate management of the huge amount of genomic data, an excellent knowledge of the biological model systems and of the biodiversity, and, most importantly, in a critical analysis of the inference and in an imaginative proposal of alternatives. The articles gathered in this special issue provide a good illustration, with software development [8,9], model and method development [10-13], and their applications [14-21]. The rest of this introduction article gives an overview of these papers.

## Organismal phylogenetic inference and evolutionary models

One of the challenging aspects of inferring a species phylogeny from genomic data is the selection of orthologous sequences. Roure et al. [9] develop SCaFoS, a software to help handling large alignments of putatively orthologous sequences. Several options are provided to detect and discard in-paralogs and out-paralogs. The software is aimed at maximizing the amount of phylogenetic signal in the resulting alignment by minimizing the amount of missing data and by selecting, when possible, the slowest evolving sequence.

Sanderson and McMahon [10] take a completely different perspective and infer the organismal phylogeny using genes with numerous duplications. The key principle is to use the ancient, but rarely used, gene tree parsimony method [22] to infer species phylogeny. Interestingly, Sanderson and McMahon obtained a phylogeny that is in excellent agreement with the expected one, although they used mainly ESTs data that yield a very incomplete sample of paralogs, demonstrating the potential power of this approach.

Although Sanderson and McMahon avoid orthology inference problems, they are nevertheless confronted to the limitations of tree reconstruction method, as demonstrated by contradiction between their parsimony and likelihood inferences. Lartillot et al. [14] address the issue of systematic errors, which is particularly important in phylogenomics [23]. They convincingly demonstrate that multiple substitutions, which are the very cause of systematic errors, are severely underestimated by standard models that assume the same evolutionary process at every position (e.g. the WAG model [24]).

Along the same line, Bao et al. [13] discuss the important issue of model selection in the presence of sites partitioned in different rate classes. Codon substitution models can be described at varying levels of parameterization; how does one choose the right level for each category of sites? The authors describe a backward-elimination procedure for selecting the model parameters that is shown to be preferable to the Akaike Information Criterion and its variants. The hypothesis testing procedure they describe will prove useful in the analysis of multi-gene families.

Emphasizing again the importance of more sophisticated evolutionary models, Wang and Hickey [20] perform a detailed analysis of the codon usage in *Oryza*. Its properties appear to be very different from the ones from *Arabidopsis*. In particular, the heterogeneity of codon usage is much larger in rice. This demonstrates that codon usage can vary rapidly over rather short evolutionary periods, potentially constituting a major challenge to existing codon models. This study strongly suggests that non-stationary models need to be introduced in this field.

## Horizontal gene transfer

An important limitation in the inference of the organismal phylogeny is the occurrence of horizontal gene transfers (HGT). Using a well-studied set of 13 Proteobacteria, Comas et al. [18] look for the categories of genes that are the most likely to contain vertical signal (i.e. phylogenetic signal derived through inheritance rather than HGT) and show that essential genes, but also many poorly characterized ones, carry the most such signal. This study demonstrates that factors determining success of HGTs are far from being fully understood.

Marri et al. [19] look at the problem at a very short evolutionary scale, i.e. closely related Corynebacteria. A maximum likelihood inference using a gene insertion/deletion model [25] demonstrates that most of the acquired genes are rapidly lost. This study thus demonstrates that the simple observation of very different gene complements among closely related strains does not prove that fixed HGTs are common in Bacteria.

## Detecting Darwinian selection

One of the most promising uses of phylogenetic approaches in genomics is the possibility to detect Darwinian selection. Chen and Blanchette [12] take an original view on Darwinian selection. Instead of looking at selection at the amino acid level as usually done, they introduce a new model to find nucleotides that are unexpectedly conserved, given known constraints at the amino acid level. The application of their method to complete mammalian genomes will contribute to the understanding of the many non-coding constraints acting at the mRNA level.

Phylogenetic footprinting approaches have originally focussed on the identification of regions that have been under negative selection throughout a given phylum [26]. However, heterotachy, i.e. variation of the evolutionary rate of a given position over time, is now commonly used as a source of functional information. Sites that change their rates after gene duplication are likely to be involved in function shift. Dorman [11] proposes a Bayesian method to simultaneously detect the branch along which such rate shifts occurred and the sites that are affected and use it to identify such a shift at the divergence of B and C subtypes of HIV.

Detecting Darwinian selection remains a difficult task and therefore the in-depth study of well-defined cases is still required to validate future methods that will be used at large scale. Weadick and Chang [27] select the guppy, *Poecilia reticulate*, because its visual system is under strong sexual selection. They show that long-wavelength sensitive opsins are highly duplicated in this fish and several sites are under positive selective pressure. A more detailed functional characterisation of these proteins, in particular with respect to mate preference adaptation, is therefore of prime importance and will provide an ideal case study for all bioinformatics methods aimed at detecting Darwinian selection.

## Homology detection

One of the original phylogenomics applications was toward the functional annotation of proteins based on their phylogenetic relationships to other annotated proteins from the same family [1]. Two aspects of this problem were studied here: (1) the identification of global functional homologs in families of multi-domain proteins [8], and (2) the identification of remote homologs [21]. More precisely, Krishnamurthy et al. [8] show that even phylogenetically-informed approach can, in some cases, be misleading, especially for multi-domain proteins. The authors introduce FlowerPower, a program and web server for the detection of global homologs, and show that it identifies functional homologs more consistently than competing approaches.

Woodhams et al. [21] illustrate one of the major limitations of comparative sequence analysis, that is the extreme difficulty in detecting distant homolog. This issue is often wrongly overlooked, even when the presence or absence of a gene in a given genome is crucial for the analysis. Using secondary structure and promoter region analysis, homologs of RNase MRP are discovered in almost all the eukaryotes for which complete genomes are available. This demonstrates that RNase MRP was likely present in the most recent common ancestor of eukaryotes, contrary to previous hypotheses. This study stresses the urgent need to improve methods to detect remote homologs, especially for studying the evolution of genome content.

## Applications of phylogenomics to detailed biological questions

Phylogenomics has the potential of testing specific biological hypotheses and identifying interesting and unexpected evolutionary processes. Analyzing human tiling array data, Zhang et al. [15] use a simple phylogenetic analysis to identify a number transcripts that are only conserved within primates, thus illustrating the limitations of phylogenetic footprinting approaches based on sequence conservation within larger sets of species. Many of these transcripts exhibit a stable secondary structure and are thus strong candidates for primate-specific non-coding RNAs.

Smith et al. [16] study the evolution and positional distribution of the important cycling-AMP response elements among animal promoter sequences. The binding sites show a strong positional preference in vertebrates, where such a bias is absent in non-vertebrate species. The authors study substitution rates inside functional binding sites and show that a significant number of them are affected by substitutions due to CpG deamination. Improving our understanding of the mutational processes in non-coding functional regions like transcription factor binding sites is critical to improve their computational identification.

## Evolution of protein-protein interaction network

Although the sequencing of complete genomes allows a comprehensive view on the genetic material of an organism, it is challenging to take into account simultaneously the evolutionary history and the multiple interactions that make an organism. Pagel et al. [17] take a first step in this direction by studying the evolution of protein-protein interaction networks in fungi. The preferential attachment hypothesis (i.e. new proteins in a network tend to interact with proteins already having numerous interactions) is generally accepted to explain the scale free nature of interaction networks. However, Pagel et al. find no evidence in favour of this hypothesis and instead demonstrate that a simple model in which proteins differ in their propensity to form attachments explains well their data. Although the small size of the data set analyzed limits the conclusions of the study, the quality of the phylogenetic method will certainly set a standard for future works.

## Perspectives

As can be seen by this collection of articles, phylogenomics is an extremely diverse and promising field. We emphasize that although the availability of larger and larger amounts of data promises an increased accuracy in phylogenomic analyses, it does not reduce the need for sophisticated and rigorous methods based on more accurate models of evolution. We hope that this meeting and this special issue will help researchers take advantage of both the genomic and phylogenetic approaches of phylogenomics and further enrich this domain by integrating new views. We are hoping to make this a regular gathering that will seed the synergy required for the field to achieve its full potential.
